# Possible Relevance of Soluble Luteinizing Hormone Receptor during Development and Adulthood in Boys and Men

**DOI:** 10.3390/cancers13061329

**Published:** 2021-03-16

**Authors:** Li Juel Mortensen, Mette Lorenzen, Anne Jørgensen, Jakob Albrethsen, Niels Jørgensen, Søren Møller, Anna-Maria Andersson, Anders Juul, Martin Blomberg Jensen

**Affiliations:** 1 Group of Skeletal, Mineral and Gonadal Endocrinology, University Department of Growth and Reproduction, Rigshospitalet, 2100 Copenhagen, Denmark; li.juel.mortensen@regionh.dk (L.J.M.); mette.lorenzen.01@regionh.dk (M.L.); 2 Department of Growth and Reproduction and International Center for Research and Research Training in Endocrine Disruption of Male Reproduction and Child Health (EDMaRC), Rigshospitalet, University of Copenhagen, Blegdamsvej 9, 2100 Copenhagen, Denmark; Anne.Joergensen.02@regionh.dk (A.J.); Jakob.Christian.Albrethsen@regionh.dk (J.A.); Niels.Joergensen@regionh.dk (N.J.); Anna-Maria.Andersson@regionh.dk (A.-M.A.); Anders.Juul@regionh.dk (A.J.); 3 Center for Functional and Diagnostic Imaging and Research, Department of Clinical Physiology and Nuclear Medicine 260, Hvidovre Hospital, 2650 Copenhagen, Denmark; Soeren.Moeller@regionh.dk; 4 Department of Clinical Medicine, Faculty of Health Sciences, Copenhagen University, 2200 Copenhagen, Denmark; 5 Division of Bone and Mineral Research, Harvard School of Dental Medicine/Harvard Medical School, Boston, MA 02115, USA

**Keywords:** LH receptor, puberty, development, infertility, extra-gonadal effects, gonadotropins, fetal kidney, fetal adrenal gland, TCAM2, NTera2

## Abstract

**Simple Summary:**

The reproductive hormones luteinizing hormone (LH) and human chorionic gonadotropin (hCG) are both agonists for the luteinizing hormone receptor (LHCGR) and essential for male reproduction during development and adulthood. LHCGR is expressed and stimulates testosterone production from the testicular Leydig cells. In this study, we demonstrate the presence of soluble LHCGR in blood, urine, and seminal fluid in both healthy boys and men, and patients with aberrations in sex-chromosomes. We show how circulating levels of sLHCGR are associated with pubertal development, testicular function, and semen quality and demonstrate that LHCGR is released from fetal human non-gonadal tissue. sLHCGR is released into serum by testis and other organs, which suggests possible extra-gonadal effects of LH or hCG in boys and men.

**Abstract:**

Luteinizing hormone (LH) and human chorionic gonadotropin (hCG) are agonists for the luteinizing hormone receptor (LHCGR) which regulates male reproductive function. LHCGR may be released into body fluids. We wish to determine whether soluble LHCGR is a marker for gonadal function. Cross-sectional, longitudinal, and intervention studies on 195 healthy boys and men and 396 men with infertility, anorchia, or Klinefelter Syndrome (KS) were used to correlate LHCGR measured in serum, seminal fluid, urine, and hepatic/renal artery and vein with gonadal function. LHCGR was determined in fluids from in vitro and in vivo models of human testicular tissue and cell lines, xenograft mouse models, and human fetal kidney and adrenal glands. Western blot showed LHCGR fragments in serum and gonadal tissue of similar size using three different antibodies. The LHCGR-ELISA had no species cross-reactivity or unspecific reaction in mouse serum even after human xenografting. Instead, sLHCGR was released into the media after the culture of a human fetal kidney and adrenal glands. Serum sLHCGR decreased markedly during puberty in healthy boys (*p* = 0.0001). In healthy men, serum sLHCGR was inversely associated with the Inhibin B/FSH ratio (β −0.004, *p* = 0.027). In infertile men, seminal fluid sLHCGR was inversely associated with serum FSH (β 0.006, *p* = 0.009), sperm concentration (β −3.5, *p* = 0.003) and total sperm count (β −3.2, *p* = 0.007). The injection of hCG lowered sLHCGR in serum and urine of healthy men (*p* < 0.01). In conclusion, sLHCGR is released into body-fluids and linked with pubertal development and gonadal function. Circulating sLHCGR in anorchid men suggests that sLHCGR in serum may originate from and possibly exert actions in non-gonadal tissues. (ClinicalTrials: NTC01411527, NCT01304927, NCT03418896).

## 1. Introduction

Luteinizing hormone (LH) and human chorionic gonadotropin (hCG) are both agonists for the luteinizing hormone receptor (LHCGR) and essential for male reproduction during development and adulthood [1]. LHCGR is a G protein-coupled receptor known to be expressed in Leydig, granulosa-lutein, and theca cells and is a potent regulator of sex steroid production [1]. hCG is a potent and long-lasting agonist but is only of physiological importance during fetal development [2]. hCG is not produced during adulthood, except for some germ cell and pancreatic cancers, although an older study found low pulsatile secretion of pituitary-hCG in adult men and women [3]. hCG is used therapeutically in women to induce ovulation during fertility treatment and in men to stimulate testosterone synthesis and potentially spermatogenesis in men with hypogonadotropic hypogonadism or as a diagnostic test for endogenous testosterone production [4]. Ablation of the LH receptor (LHCGR) in mice leads to a dramatic decrease in testosterone and semen quality [5], and several groups have identified mutations and SNPs in the LHCGR demonstrating its importance for reproductive functions in humans [6,7,8].

The gene for LHCGR is located on chromosome 2 and comprises 11 exons, of which exons 1–10 encode the extracellular domain, while exon 11 encodes the seven-transmembrane and the intracellular domain [9]. Several isoforms of LHCGR exist and it has been suggested that one isoform is secreted because it lacks the transmembrane anchor [10]. Another possibility is that the release of LHCGR into body fluids is dependent on enzymatic cleavage at the cell membrane by proteins such as ADAMs, MMPs, and others known to cleave transmembrane proteins. In fact, hCG has been shown to specifically upregulate MMP-2 and MMP-9 [11,12] and several studies have shown post-transcriptional regulation of LHCGR [13,14]. Soluble LHCGR (sLHCGR) has been measured by an ELISA platform in both serum and follicular fluid from women [15,16]. The specificity of the antibodies used in the ELISA assay were validated by expressing different LHCGR fragments in CHO cells for epitope mapping and by examining expression in the human placenta [15,16,17]. Moreover, sLHCGR has been measured in serum by LC-MS/MS [18]. Female serum concentrations of sLHCGR have been linked with implantation rate in fertility treatment, preeclampsia, preterm birth, and pregnancy carrying a Down’s syndrome child [15,16], but have so far not been validated by other groups. sLHCGR has to our knowledge never been described in body fluids from healthy men, boys during pubertal development, or men with sex-chromosome aberrations. The putative function of sLHCGR in the circulation of men may be to transport or modify the activity of LH or hCG by high-affinity binding and thereby be a marker of gonadal function.

## 2. Materials and Methods

### 2.1. Cohorts

#### 2.1.1. Young Men from the General Population

In Denmark, all young healthy men of 18 years are required to undergo a physical examination to determine if they are fit for military service. Since 1996 approximately 300 conscripts representing the general population have annually been examined to determine their semen quality. They all delivered a semen sample, filled in a questionnaire, had an ultrasound examination of the testicles performed and a blood sample drawn [19]. Biobanked serum- and semen samples, as well as ultrasonic evaluations and information on BMI and age from 148 men investigated in 2013 and 2014, were included in the current study (Ethical committee approval: H-KF-289428).

#### 2.1.2. Pre- and Post-Pubertal Healthy Boys

The Copenhagen puberty study is a combined cross-sectional and longitudinal study of healthy Danish children [20]. From randomly selected public schools, 6203 children were invited to participate. The total number of participants was 2020 children. Every six months, 209 children (101 boys) were followed longitudinally with the physical examination, evaluation of pubertal stage by Tanner, and blood and urine samples. Thirty-six of the boys who followed longitudinally were included in this present study to evaluate sLHCGR during puberty (Clinical trials: NTC01411527).

#### 2.1.3. Infertile Men without Serious Comorbidity

The Copenhagen-Bone-Gonadal Study is a cohort of 307 infertile men with no serious co-morbidities referred from 2011 to 2015 to our andrological outpatient clinic and included a total of 1427 men [21,22]. All men were referred to our clinic due to low semen quality and a wish of obtaining parenthood. All had a physical examination including an ultrasound examination of testicles performed, a blood sample drawn, and they delivered semen samples. A specialized physician obtained their detailed patient history. The men were followed for 150 days and treated with either vitamin D or placebo as described previously [21]. All the presented data in this study are from their initial visit prior to the intervention and all included variables were predefined (Clinical Trials: NCT01304927).

#### 2.1.4. Men and Boys with Klinefelter Syndrome and Patients with Anorchia

We identified 64 boys and men from our andrological out-patient clinic with Klinefelter syndrome (KS), 47 were post-pubertal and 16 were pre-pubertal, defined by Tanner staging. We had information on exact chromosomal aberration, patient history, status on the pubertal onset, and available serum on all. Seven boys have followed longitudinally prior to and during pubertal onset with regular clinical evaluation and blood sampling.

We also included eight men with normal chromosome numbers who had both testicles removed due to previous conditions: testicular germ cell cancer (*n* = 4), disseminated prostate cancer (*n* = 1), bilateral cryptorchidism and childhood orchiectomy (*n* = 1), bilateral Germ Cell Neoplasia in Situ (*n* = 1), and orchiectomy based on trauma with later testicular cancer in the remaining testes (*n* = 1).

#### 2.1.5. Adults Undergoing Splanchnicus Flow Measurement

We included three men and three post-menopausal women with normal liver function who were admitted under suspicion of mesenteric ischemia and referred for measurement of splanchnic blood flow but turned out to be without mesenteric ischemia. Blood samples were collected simultaneously from the hepatic, renal, and femoral veins and from the corresponding artery (Ethical committee approval: H.18048245)

#### 2.1.6. Intervention Study: Healthy Men Exposed to Human Chorion Gonadotropin

Eleven healthy men were recruited to participate in a clinical intervention through bulletins at Rigshospitalet, Denmark. The volunteers were treated with 5000 IU human chorion gonadotropin and their maximum endogenous testosterone production was tested by comparing baseline blood and urine- samples with subsequent sampling collected 8, 24, and 72 h after hCG injection (Clinical Trials: NCT03418896).

### 2.2. Mice, Human Tissue, and Cell Lines

Two human testicular cancer cell lines NTera-2 and TCam-2 representing embryonal carcinoma and seminoma respectively were used and cultured as described previously [23].

Serum was collected from nude mice without tumors (WT) and TCam-2 xenograft nude mice treated with vehicle, LH, or hCG (Danish Animal Experiments Inspectorate Copenhagen, Denmark, License no. 2012-15-2934-00051).

First-trimester human fetal tissue was collected from women donating the tissue after a scheduled provoked abortion. Fetal kidney and fetal adrenal gland tissue were cultured in a hanging drop model, described in detail previously, and media was collected for analyses [24,25] (Ethical committee approval: H-1-2012-007).

Adult human testis specimens were obtained from orchidectomy of men with testicular cancer (Ethical committee approval: H-1-2012-007). The tissues surrounding the tumor containing non-malignant areas were stored at −80°C or fixed overnight at 4 °C in formalin or modified Stieve’s-fixative (200 mL 37% Formaldehyde, 40 mL acetic acid added to 1 L of 0.05 M phosphate buffer, pH 7.4).

Serum from three pregnant women, approximately week 20 (*n*:2) and 28 (*n*:1) of gestation, as well as serum from two men with testicular germ cell cancers (1 with seminoma, 1 with non-seminoma), were obtained through our out-patient clinic (Permit No:KF 02-125/95; KF 01 2006-3472).

Adult human ovary RNA and adult human epididymis RNA for PCR analyses we purchased from Ambion Inc. and BioChain, respectively.

### 2.3. Biochemical Analyses

Total sLHCGR in serum, seminal fluid, urine, and various culture media was measured, during validation all was measured in duplicates with an ELISA kit from Novel Biomarkers Catalyst Lab (NBCL), Holland using antibody LHR029 raised against human LHCGR. The ELISA from NBCL had a limit of detection of 0.01 pmol/mL, and an upper detection level of 15.55 pmol/mL. Six men from the general population had sLHCGR levels higher than the upper detection limit and their samples were diluted, remeasured, and showed levels up to 30 pmol/mL. The performance of the ELISA platform has been described in detail previously [15,16,17].

We also set up two different in-house ELISAs, build by technicians in our laboratory, for which all further reagents were made available by Origin Biomarkers Ltd, the UK based on their suggested setup [26]. First, circulating sLHCGR bound to hCG was measured using a sandwich ELISA kit, with antibodies 5A10C9 (ProMab) and AM00904PU-N (OriGene) targeting LHCGR and hCG respectively. Second, total sLHCGR in arterial and venous samples from liver, kidney, and lower extremities in the six adults who had undergone splanchnicus flow measurement were likewise measured using an in-house ELISA, where we used the same antibody LHR029 (NBCL Holland) targeting sLHCGR. Two different protocols were tested [26]. All sLHCGR measurements presented in this manuscript were measured using NBCL ELISA, except hCG-sLHCGR complex and arterial/venous samples from organs, as described above. In the healthy boys, testosterone was analyzed with a DPC coat-A-count RIA kit (Diagnostic Products, Los Angeles, CA) with a limit of detection (LOD) 0.23 nmol/L and CV of 8.6% and SHBG were measured using a time-resolved immunofluorescence assay (Delfia; Wallac, Turku, Finland) with a detection limit of 0.20 nmol/liter and CV of 6.4%. In the adults, testosterone, as well as sex hormone-binding globulin (SHBG), were analyzed using chemiluminescent immune assays (Access, Beckman Coulter, USA) with LOD of 0.35 nmol/L and 0.33 nmol/L. Estradiol levels were analyzed in all samples by radioimmunoassay (Pantex, Santa Monica, CA, respectively) with a CV of 13% and a detection limit of 18 pmol/L. Serum and urine FSH and LH levels were determined using a time-resolved immuno-fluorometric assay (Delfia; Wallac, Turku, Finland) with a detection limit of 0.06 and 0.05 IU/L respectively and CV < 5% for both. Inhibin B was measured with a specific two-sided enzyme-linked immunoassay (inhibin B genII, Beckman Coulter, USA) CV < 11%. For AMH we used the Beckman Coulter enzyme immunometric assay (Immunotech, Beckman Coulter, Marseilles, France) with CV < 8%. Finally, IGF1 and IGF-BP3 were measured using chemiluminescence assay from IDS-iSYS immunodiagnostics with a CV of 10 and 9%, respectively. Free estradiol was calculated using the Mazer constant and free testosterone (FT) was calculated by the Vermeulen formula [27,28].

#### 2.3.1. Liquid Chromatography–Mass Spectrometry (LC–MS/MS)

To increase chances of identification of sLHCGR by mass spectrometry, albumin and IgG was first depleted from serum using Pierce™ Removal Kit by ThermoFisher (Catalogue number: 89875). Next, the sample was analyzed by SDS-PAGE and stained using Coomassie (BioRad, Hercules, CA, USA #1610803) (method described in Western Blot section). Finally, selected bands were excised from the gel. Attempts were made to identify sLHCGR by trypsin digestion and LC-MS/MS by Alphalyse A/S (www.alphalyse.com, accessed on 17 June 2020).

#### 2.3.2. Semen Analysis

Healthy young men and infertile adult participants produced a semen sample and self-reported information of duration of ejaculation abstinence was obtained. Trained technicians conducted semen analysis as described in detail previously [19]. Semen volume was estimated by weighing. For sperm motility assessment, duplicates of 10 µL of well-mixed semen were placed on a glass slide, examined on a heating stage kept at 37 °C, under a microscope at × 400 magnification, and spermatozoa were classified as progressive motile (WHO class A + B), non-progressive motile (class C) or immotile (class D). The average of the two motility assessments was used. For the assessment of the sperm concentration, the samples were diluted in a solution of 0.6 moL/L NaHCO3 and 0.4% (*v*/*v*) formaldehyde in distilled water in duplicates and subsequently assessed using Bürker-Türk hemocytometer, and averages of the assessments used. Only spermatozoa with tails were counted. Finally, smears were prepared, Papanicolaou stained, and spermatozoa morphology assessed according to strict criteria [29].

#### 2.3.3. Reverse Transcriptase PCR (RT-PCR)

Total RNA was extracted and isolated from human testis tissue using a NucleoSpin RNA II purification kit (Macherey-Nagel, Düren, Germany)according to the manufacturer’s protocol (Macherey-Nagel, Cat#740955.50). RNA was reverse transcribed into cDNA using a dT20 primer and random hexamers. RPS20 was used as a reference gene. Primers for LHCGR were spanning exon 2–4 (F: CCTACCTCCCTGTCAAAGTG, R: ATGCTCCGGGCTCAATGTATC) and exon 11 (F: CGATTTCACCTGCATGGCAC, R: GTGTAGCGAGTCTTGTCTAG). Representative bands from each primer combination were sequenced for verification (Eurofins Genomics).

#### 2.3.4. Western Blotting

Human testis tissue was homogenized in lysis buffer and diluted in SDS loading buffer followed by heating at 95 °C for 5 min. Samples were loaded onto a 4–20% precast polyacrylamide gel (BioRad, cat#456-8096) and run for 1 h at 100 V to separate proteins. To detect sLHCGR in serum, albumin and IgG were removed with Pierce Albumin/IgG Removal kit (Thermo Scientific, Waltham, MA, USA, Cat#89875) before loading the samples onto the gel. After gel electrophoresis proteins were transferred to a polyvinylidene difluoride membrane using a wet blot apparatus (BioRad). Membranes were blocked for 1 h in Tris-buffered Saline with 5% nonfat dry milk, then incubated with primary antibodies at 4 °C overnight and with secondary antibodies at RT for 1 h. Membranes were developed using enhanced chemiluminescence (Thermo Scientific Super Signal West Femto Maximum Sensitive Substrate, Cat#34095) and photodetection was done with chemiDoc MP imaging system (BioRad, Cat#17001402). Three different antibodies were used to detect LHCGR in tissue and serum: Aviva System Biology (OASG04237); NBCL Holland, the same antibody as in serum ELISA kit (LHR029) and Santa Cruz Biotechnology (Dallas, TX, USA, SC-26341). Beta-2 microglobulin was used as a loading control.

#### 2.3.5. Immunohistochemistry

Immunohistochemistry was conducted as previously described [25]. In brief, sections were fixed in modified Stieve’s fixative, deparaffinized, and rehydrated. Antigen retrieval was done in a pressure cooker with citrate retrieval buffer (10 mM, pH 6.0). Endogenous peroxidase was blocked with 3% (*v*/*v*) H_2_O_2_ in methanol for 30 min. Between each step, sections were washed in TBS. Sections were incubated with 0.5% BSA horse serum for 30 min, followed by incubation with two different primary antibodies overnight and 1 h at room temperature (LHR029 as in WB/ELISA, and Santa Cruz Biotechnology SC-25828). Sections were incubated with secondary antibodies for 30 min. Visualization was performed with aminoethyl carbasole (AEC, Invitrogen by Life Technologies/Thermo Fisher Scientific, Waltham, MA, USA. Negative controls were without primary antibodies. None of the negative controls showed staining. Counterstaining was performed with Mayer’s hematoxylin and mounting was done with Aquatex. Sections were evaluated on a Nikon Microphot-FXA microscope (Nicon Inc., Melville, NY, USA). A final analysis was done using the software NDPview version 1.2.36 (Hamamatsu Photonics, Iwata Ciy, Japan.

### 2.4. Statistical Analyses

Descriptive statistics were calculated for all variables and presented as the mean with standard deviation. Associations between sLHCGR in serum and seminal fluid and outcome variables (hormones, pubertal onset, testicular size, age, and BMI) were conducted using regression analyses. Gaussian distribution of numerical variables was evaluated by PP-plots of residuals to secure the validity of the regression models. As a result, sLHCGR in serum and urine was logarithmically transformed when used as the dependent variable in all cohorts as well as sLHCGR in seminal fluid from the young healthy men and seminal/serum sLHCGR-ratio in the pooled analyses. Measurements of hormones and sLHCGR below the limit of detection were set to a value of half of the detection limit. In the large cohorts, sLHCGR was further split into quartiles and Kruskal-Wallis was performed with Dunn-Bonferroni post hoc test, to adjust for multiple comparisons. Longitudinal measurements during puberty, outcomes from clinical intervention, and comparison of sLHCGR in different body fluids from the same man were analyzed using Wilcoxon Paired Test. When performing more than one paired test, the level of significance was corrected for multiple testing using the Bonferroni method. The following ratios were calculated and analyzed in regression models as independent: inhibin B/FSH, testosterone/LH, testosterone/estradiol. In all prepubertal boys, analyses on testosterone and estradiol (including ratios) were left out, as all but two measurements were below the limit of detection. For all analyses, *p* < 0.05 was considered statistically significant. All presented analyses of semen quality were adjusted for the duration of ejaculation abstinence. Statistical analyses were performed using PASW GradPack 18.0 (SPSS Inc., Chicago, IL, USA). Graphs and plots were made using GraphPad Prism version 7.02 for Windows, GraphPad Software, La Jolla California USA, www.graphpad.com (accessed on 8 June 2017).

## 3. Results

### 3.1. Several Isoforms of LHCGR Is Expressed in Testis and Detected in Serum

**Figure 1:**
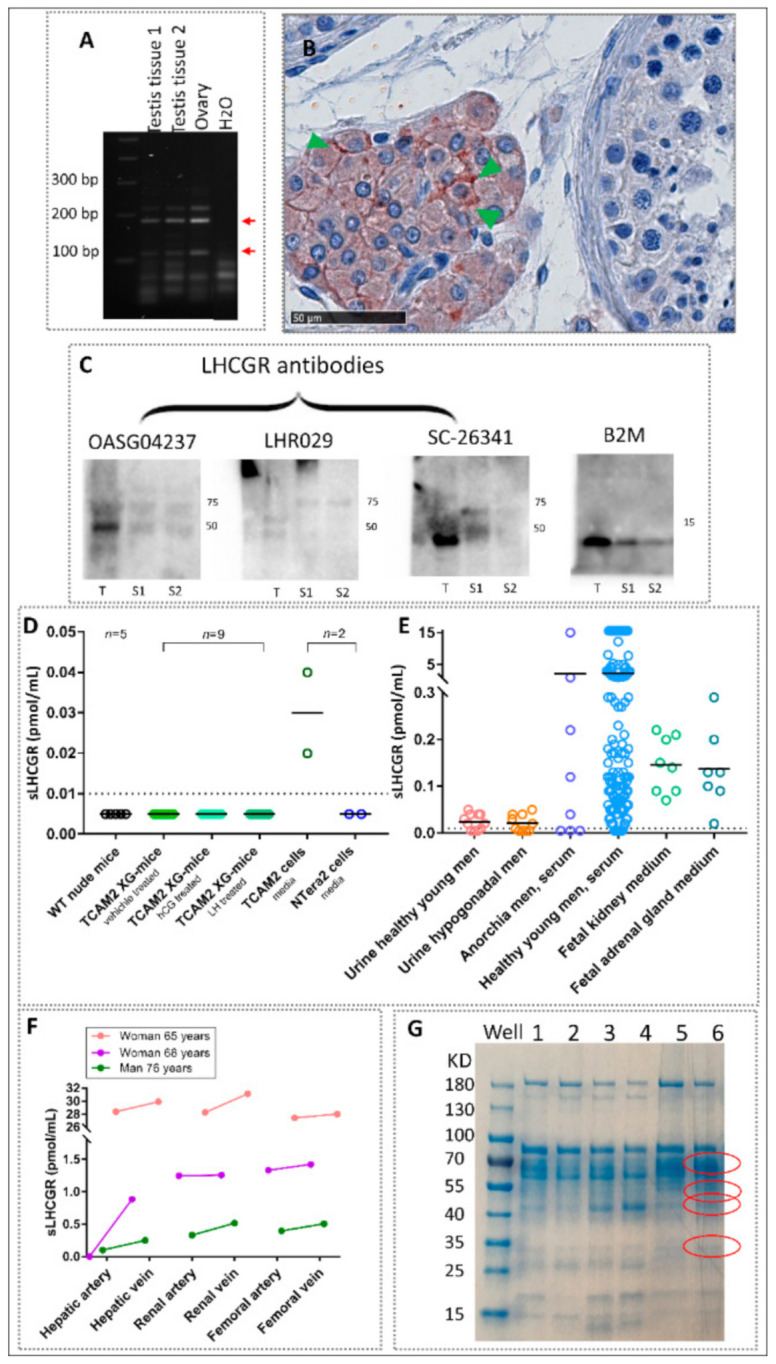
In vitro and baseline analyses of luteinizing hormone receptor (LHCGR). (**A**). Reverse Transcriptase PCR (RT-PCR) with a primer spanning exon 2–4 of LHCGR in two testis tissue and one ovarian tissue sample. Two bands (red arrows) of approximately 100 and 200 bp were sequenced and identified as LHCGR isoforms. (**B**) Immunohistochemistry of human adult testis tissue stained with LHR029 antibody from the ELISA kit showing LHCGR expression in Leydig cells (green arrows). Image is from × 40 magnification (**C**): Western Blot detecting LHCGR with three antibodies targeting different domains examined on testis tissue (T), and in serum from men with high Soluble LHCGR (sLHCGR) level (S1) and low sLHCGR level (S2). (**D**) Human sLHCGR is unmeasurable in serum from WT nude mice, in xenograft (XG) mice implanted with a human seminoma cell line (TCam2), treated with either vehicle, hCG, or LH. sLHCGR is measurable in low levels in media from TCam2 cells but not in a human embryonic carcinoma cell line, NTera2. (**E**) sLHCGR present in urine at comparable levels in healthy and hypogonadal men. sLHCGR measurable in serum from 8 men with both testicles removed at comparable levels as in healthy young men. sLHCGR measurable in media from the cultured human fetal adrenal gland and human fetal kidney. (**F**) Total sLHCGR from arterial- and venous phase across liver, kidney and lower extremity measured in one man and two post-menopausal women shows no significant difference in arterial and venous levels, independent of the organ. (**G**) SDS-protein gel electrophoresis of human serum samples after Immunoglobulin and albumin removal from left well 1–6: serum from 2 normal men, 1 man with testicular germ cell cancer, seminoma, 1 man with testicular germ cell cancer, non-seminoma, and 2 pregnant women. Four bands of approximately 35, 50, 60, and 75 kDa from the pregnant woman in well number 6 were analyzed by LC-MS/MS.

Several LHCGR isoforms were identified in reproductive tissues by RT-PCR using primers spanning exon 2–4. Two strong bands at approximately 100 and 200 base pairs were detected in human testis samples and ovary RNA and confirmed to be LHCGR by direct sequencing (Figure 1A and Figure S1B). Exon 11 was also expressed in the testis and ovary (Figure S1B). By western blot (WB) the OASG04237 antibody, targeting the intracellular part, we measured LHCGR in human testis tissue and in two serum samples; one serum sample from a man with high sLHCGR measured by ELISA and one serum sample from a man with low sLHCGR. WB showed two bands of 50 and 75 kDa in the testis and weaker bands of similar size in both serum samples and a week band around 75 kDa predominantly in the serum samples (Figure 1C). Two bands at 50 and 75 kDa were also found using the LHR029 antibody, which binds to an extracellular part of LHCGR, although with lower intensity in both serum samples compared with testis and again a robust detection of a 75 kDa band in the serum samples. The SC-26341 antibody, detecting an unknown part of the extracellular domain of LHCGR showed bands around 50 and 75 kDa in all samples while the 75 kDa band was much stronger in the testis compared with serum samples. Noteworthy, the level of the control beta-2 microglobulin was as expected markedly lower in the serum compared with testis tissue, which hampers the quantitative use and comparison between LHCGR levels in tissue and serum. Densitometry readings of bands and uncropped blots are shown in Figure S2. Immunohistochemistry using the same antibody as in the ELISA assay (LHR029) on testicular tissue from an adult man with Germ Cell Neoplasia in Situ and mild Leydig cell hyperplasia showed a combined membrane-bound and slight cytoplasmic expression of LHCGR in most Leydig cells (Figure 1B and Figure S1A with antibody from Santa Cruz).

### 3.2. sLHCGR Is Detectable in Body Fluids but Undetectable in Serum from Xenograft Mice

There was no detectable sLHCGR in serum from nude mice with or without TCam-2 xenograft tumors treated in vivo with either vehicle, LH, or hCG (Figure 1D).

However, media from cultured TCam-2 cells showed a low but detectable level of sLHCGR (Figure 1D), which combined proves that the human ELISA does not crossreact with any mouse proteins available in serum. We also did not detect sLHCGR in the unconditioned culture media containing fetal bovine serum.

sLHCGR was detectable and available at comparable levels in urine from healthy and hypogonadal men (mean 0.024 pmol/mL vs. 0.021 pmol/mL). Urine levels were on average approximately more than 100-fold lower than in serum (mean 2.21 pmol/mL and hypogonadal mean 1.80 pmol/mL) suggesting that renal elimination is independent of hypogonadism and not influenced by more than 10 years age difference (Figure 1E). Moreover, the low concentration in urine suggests that sLHCGR like other proteins is not filtrated or excreted in quantities that exceed the renal reabsorption capacity. Noteworthy, sLHCGR in serum from eight men who had *both* testicles removed showed average concentrations in serum at a comparable level to the young men (mean 2.04 pmol/mL vs. 2.21 pmol/mL), (Figure 1E) and hypogonadal men (mean 2.04 pmol/mL vs. mean 1.80 pmol/mL). Furthermore, human fetal adrenal gland and human fetal kidney tissue showed detectable levels of sLHCGR (mean 0.15 pmol/mL, mean 0.14 pmol/mL respectively) (Figure 1E), and thus higher levels than sLHCGR released from TCam-2 cells in vitro (mean 0.03 pmol/mL) (Figure 1D). Total sLHCGR in sera from 3 men and 3 postmenopausal women were measured from both the arterial- and venous phase across the liver, kidney, and lower extremities. One man and two women had levels above the limit of detection (Figure 1F) whereas the remaining had undetectable levels in all samples. We did not detect any significant difference in sLHCGR from the arterial to the venous side of any organ and thus excluding these organs as the site of origin (*t*-test: *p* values all > 0.31).

### 3.3. LC-MS/MS on Human Serum

Serum from normal men (*n*:2), testicular germ cell cancer patients (1 seminoma, 1 non-seminoma), and pregnant women (*n*:2) was used for protein gel electrophoresis after albumin and globulin depletion. 4 bands of 75, 60, 50, and 35 kDa was extracted and analyzed by low sensitivity LC-MS/MS (Figure 1G) from pregnant serum because the strongest signal was found here. No tryptic peptides from LHCGR were detected by LC-MS/MS in any of the analyzed samples. The most abundant peptides despite IgG and albumin removal were albumin, complement C3, alpha-1-antitrypsin, and Vitamin-D Binding Protein (see supplementary table for all detected proteins), all the detected proteins were high abundant plasma proteins.

### 3.4. Serum sLHCGR. Diurnal Variation, Stability, Reproducibility, Freeze, and Thaw

**Figure 2:**
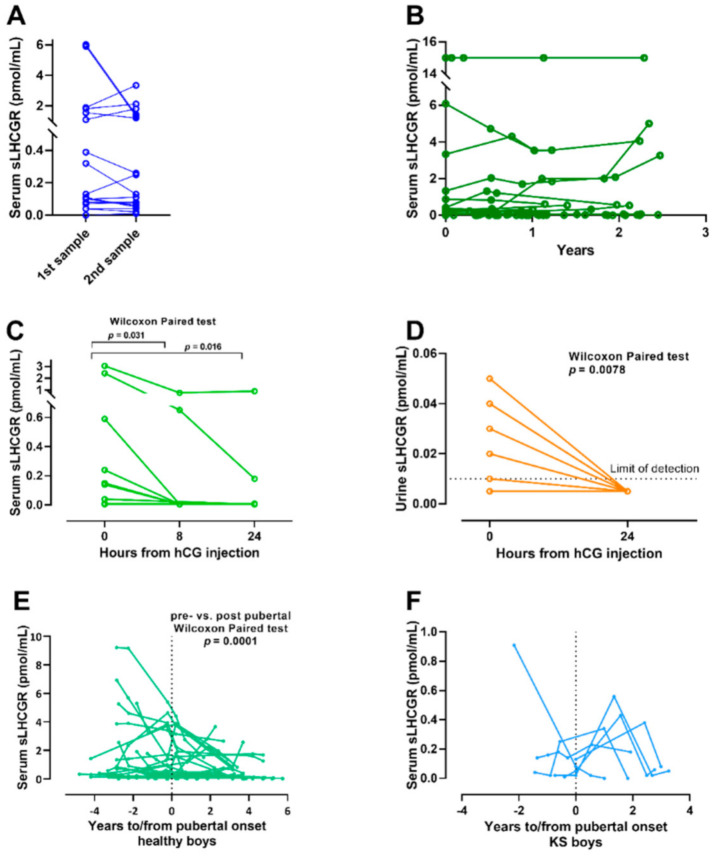
Longitudinal measurements of sLHCGR in serum and urine. (**A**): Repeated measurements of serum sLHCGR in adult Klinefelter men (timespan 4–36 months). (**B**): Repeated measurements of serum sLHCGR in 23 orchiectomized men. (**C**): Change in serum sLHCGR compared with baseline in 11 healthy men injected with 5000 IU hCG. (**D**): Change in urine sLHCGR in 11 healthy men injected with 5000 IU hCG. (**E**): Longitudinal measurements of sLHCGR in healthy boys during puberty. (**F**): Longitudinal measurements of sLHCGR in boys with Klinefelter syndrome (KS) during puberty.

We used three different lots of LHCGR Kits from the NBCL ELISA assay with a CV of 16 and 19% at the level of 0.4 and 1.7 pmol/mL. Serum-, seminal- and urine levels of sLHCGR in all cohorts are presented in Table 1, Table 2 and Table 3. Season of the year or time of the day of blood-sampling had no impact on sLHCGR levels (neither seminal nor serum levels). Intra-individual variation was low in 18 adult men with Klinefelter syndrome with consecutive measurements of sLHCGR for 4–40 months (Figure 2A). All men except two had stable levels of sLHCGR. In addition, consecutive measurements on 23 orchiectomized men followed for 16–30 months showed that only one man had a marked increase during this period, while the remaining 22 men overall had no differences in serum sLHCGR levels (Figure 2B). Multiple Freeze and thaw decreased sLHCGR concentration by ~40%.

### 3.5. Serum and Urine LHCGR Are Influenced by Treatment with Human Chorion Gonadotropin

Injection of 5000 IU hCG (see Table 1 for baseline characteristics) lowered serum sLHCGR after 8 h (Wilcoxon Paired test *p* = 0.031) and the decrease was augmented after 24 h (Wilcoxon Paired Test *p* = 0.016, after Bonferroni correction, *p* < 0.025) (Figure 2C). Only two men had detectable sLHCGR in serum 24 h after injection those with the highest baseline values. The fast effect of hCG on circulating sLHCGR levels was also evident in the urine as the urinary sLHCGR levels fell to below the limit of detection in all the men after 24 h (Wilcoxon Paired Test *p* = 0.0078) (Figure 2D). Baseline serum sLHCGR, prior to hCG injection, was not correlated with baseline urine-sLHCGR, serum testosterone nor serum LH level, or with delta serum testosterone nor delta serum LH. Neither urinary sLHCGR was correlated with age nor reproductive hormones in serum. hCG induced an increase in serum testosterone, from 19.6 nmol/L (3.3 SD) to 28.2 nmol/L (4.4 SD) after 24 h (Wilcoxon Paired Test: *p* = 0.001) and to 43.9 nmol/L (8.6 SD) after 72 h (*p* = 0.001) (Figure S3A), while LH decreased significantly after 24 h (Wilcoxon Paired Test: 8 h *p* = 0.43, 24 h *p*= 0.001, 72 h *p* = 0.001) (Figure S3B).

### 3.6. Longitudinal Measurement in Children Show a Decline in sLHCGR during Puberty Only in Healthy Boys

To determine how pubertal onset with increasing gonadotropin secretion influence sLHCGR, 36 normal boys were followed longitudinally, and pubertal onset was evaluated by Tanner staging (baseline characteristics are presented in Table 2).

Individual post-pubertal levels of sLHCGR were significantly lower than the prepubertal levels (pre- vs. postpubertal Wilcoxon Paired Test: mean (1.36 (2.2 SD) pmol/mL vs. 0.60 (0.89 SD) pmol/mL *p* = 0.0001) (Figure 2E). This supports that sLHCGR may decrease in response to the increase in serum gonadotropins, but neither reproductive hormone levels nor BMI, age, or testicular size were associated with sLHCGR at any timepoint during pubertal development. In accordance, we found no associations between sLHCGR and urinary LH or FSH prior to or after puberty. For comparison, seven KS boys (Baseline characteristics are presented in Table 3) were followed longitudinally without testosterone induction of puberty.

The KS boys did not show a similar decline in sLHCGR over the course of puberty as was seen in the boys without chromosomal aberrations. All KS boys (but one) experienced a peak in sLHCGR concentration at the time of pubertal onset, however not reaching statistical significance (Figure 2F). Klinefelter boys also experienced an increase in gonadotropins but for unknown reasons they did not experience the same decrease in sLHCGR, which indicates that high sLHCGR may be related to impaired gonadal function. sLHCGR in serum was slightly lower in young men with KS compared to young healthy men (0.92 pmol/mL vs. 2.21 pmol/mL, *p* = 0.031, Table 1 and Table 3). Four men with KS who presented with the highest levels of sLHCGR in serum did not differ from the remaining men with KS regarding phenotype.

### 3.7. Serum sLHCGR Is Associated with the Gonadal Marker Inhibin/FSH Ratio and Estradiol in Healthy Men

**Figure 3:**
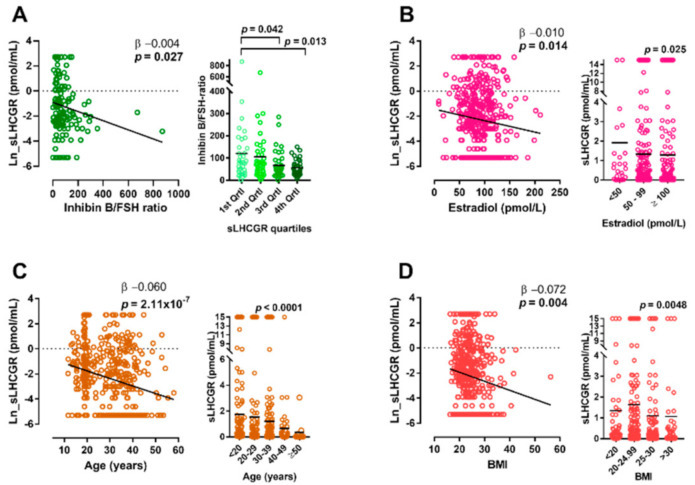
Association between sLHCGR in serum and reproductive hormones, age and BMI in adult men. (**A**): sLHCGR and gonadal function in 148 young healthy men. A1: Linear regression shows an inverse association between sLHCGR and Inhibin B/FSH-ratio. A2: Serum sLHCGR stratified into quartiles and inhibin B/FSH-ratio, bar shows mean, *p*-values adjusted for multiple comparisons using Dunn-Bonferroni post hoc test. (**B**–**D**): Serum sLHCGR in a pooled analysis of all men with normal sex-chromosomes after pubertal onset. B1: Linear regression showing an inverse association between sLHCGR and estradiol. B2: sLHCGR in all men stratified into three groups based on baseline estradiol, bar shows mean, *p*-value from Kruskal Wallis. C1: Linear regression showing an inverse association between sLHCGR and age. C2: sLHCGR in all men stratified into five groups based on age, bar shows mean, *p*-value from Kruskal Wallis. D1: Linear regression showing an inverse association between sLHCGR and BMI. D2: sLHCGR in all men stratified into four groups based on BMI, bar shows mean, *p*-value from Kruskal Wallis.

Baseline characteristics of 148 men from the general population can be found in Table 1. Serum sLHCGR was inversely associated with the gonadal marker Inhibin B/FSH ratio (β −0.004, *p* = 0.027 and removal of two far-right outliers did not change the association: β −0.003, *p* = 0.018) (Figure 3A). The following stratification into sLHCGR-quartiles, a significant downward trend was found, and men in the lowest sLHCGR quartile had significantly higher Inhibin B/FSH ratio compared with men in the highest sLHCGR quartile also after adjustment for multiple comparisons (Figure 3B). However, serum sLHCGR was not associated with any other hormone or with semen quality, age, BMI, or testicular size. For comparison, 297 infertile men without serious co-morbidities were examined cross-sectionally and their baseline characteristics are presented in Table 1. Average sLHCGR in serum was low 1.05 pmol/mL (SD 3.15) and 117 out of 297 men even had undetectable levels. We found no association between sLHCGR and inhibin B/FSH-ratio (β −0.0010, *p* = 0.28 Figure S4A) or any of the measured hormones, testicular size, BMI, or age in this cohort. When stratifying the men into sLHCGR quartiles there was no difference in the Inhibin B/FSH ratio (Kruskal-Wallis *p* = 0.56, Figure S4B). We pooled all males with normal sex-chromosomes and pubertal onset from the used cohorts. This increased the total number of cases with available serum sLHCGR (*n* = 488) increased age span (range 11.7–58.0 years) and BMI span (range 16.3–56.3). Serum sLHCGR was inversely associated with estradiol (β −0.009, *p* = 0.020), age (β −0.060, *p* = 2.11 × 10^−7^) and BMI (β −0.072, *p* = 0.004), but with none of the other hormones (Figure 3B–D). When analyzing only the healthy males from all the above cohorts, the same inverse association was found with the Inhibin B/FSH ratio (β −0.003, *p* = 0.045).

### 3.8. Seminal Fluid sLHCGR Is Associated with Semen Quality and FSH

**Figure 4:**
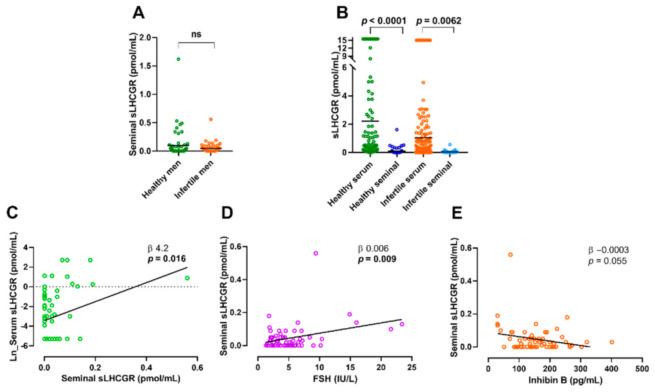
Seminal sLHCGR levels in normal and infertile men. (**A**): Seminal levels of sLHCGR in healthy and infertile men bar shows mean. (**B**): Serum vs. seminal level of sLHCGR in healthy and infertile men, bar shows mean and groups are compared using Wilcoxon Paired Test. (**C**): Linear regression showing associations between seminal and serum sLHCGR in infertile men. (**D**): Linear regression showing associations between seminal sLHCGR and serum FSH in infertile men. (**E**): Linear regression showing association between seminal sLHCGR and serum Inhibin B.

To determine whether sLHCGR was released directly into the seminal fluid a fraction of the cohorts (*n*:59 young healthy, *n*:61 infertile men) were investigated. Seminal levels of sLHCGR were comparable in normal and infertile men (Figure 4A) but remarkedly lower compared with serum levels (Figure 4B) (healthy: mean 0.10 pmol/mL (0.24SD) vs. mean 2.36 pmol/mL (4.75SD), *p* = 0.001; infertile: mean 0.05 pmol/mL (0.08SD) vs. mean 1.08 pmol/mL (3.28SD), *p* = 0.016). In the young men, no association was found between sLHCGR in seminal fluid and the investigated hormones in serum, semen parameters, age, or BMI. In the infertile men, there was a positive association between serum and seminal levels of sLHCGR (β 4.2, *p* = 0.016) (Figure 4C). Seminal fluid concentration of sLHCGR in the infertile men was positively associated with serum FSH (β 0.006 pmol/mL, *p* = 0.009) (Figure 4D) and borderline inversely associated to Inhibin B (β −0.0003 pmol/mL, *p* = 0.055) (Figure 4E).

**Figure 5:**
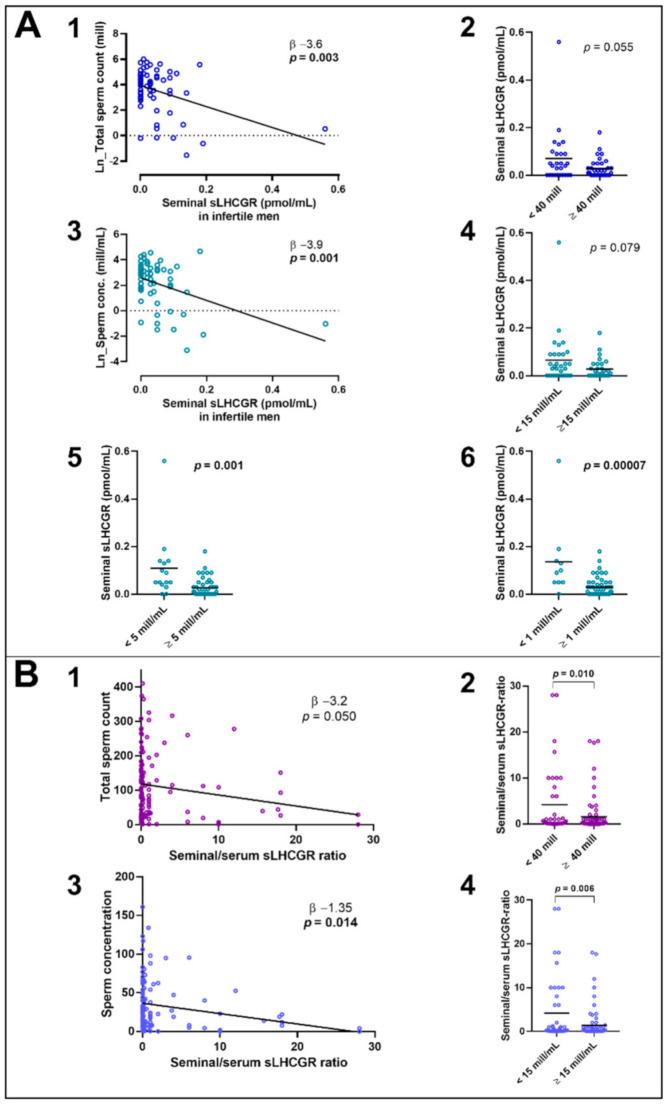
Association between sLHCGR in seminal fluid and semen quality. (**A**): Serum and seminal LHCGR concentration and semen quality in 61 men. A1: Linear regression between total sperm count and seminal sLHCGR-level. A2: Total sperm count cut-off value for low vs. normal (< 40 mill), bar shows mean. A3: Linear regression between sperm concentration and seminal sLHCGR-level. A4: Borderline significantly lower sLHCGR with decreased sperm concentration: cut-off value for low vs. normal (<15 mill/mL). A5: Significantly lower sLHCGR with decreased sperm concentration: cut-off for severe oligospermia (<5mill/mL). A6: Significantly lower sLHCGR with decreased sperm concentration: cut-off of 1 mill/mL, bar shows mean. (**B**): Pooled analyses of seminal sLHCGR data from both healthy and infertile men. B1: Linear regression between seminal/serum sLHCGR-ratio and total sperm count. B2: Significantly lower sLHCGR with decreased sperm concentration: cut-off for low vs. normal total sperm count (<40 mill), bar shows mean. B3: Linear regression between seminal/serum sLHCGR-ratio and sperm concentration. B4: Significantly lower sLHCGR with decreased sperm concentration: cut-off for low vs. normal total sperm concentration (<15 mill/mL). All seminal analyses adjusted for time of abstinence.

Moreover, seminal sLHCGR was inversely associated with total sperm count (β −3.2, *p* = 0.007) (Figure 5A) and sperm concentration (β −3.5, *p* = 0.003) (Figure 5A). The men were stratified in two groups based on sperm concentration and men with severe oligospermia (5 mill/mL) had higher seminal sLHCGR (mean 0.11pmol/mL (0.14SD) vs. 0.03 pmol/mL (0.04SD) *p* = 0.001), and particularly men with sperm concentration of less than 1 mill/mL (mean 0.14 pmol/mL (0.16SD) vs. 0.03 pmol/mL (0.04SD) *p* = 0.0002) (Figure 5A).

A pooled analysis of all seminal data showed a negative association between seminal/serum sLHCGR-ratio and total sperm count (β −3.2, *p* = 0.050, Figure 5B) and men with total sperm count <40 mill had higher seminal/serum sLHCGR-ratio than men with normal sperm count (mean 4.22 pmol/mL (7.37SD) vs. 1.56 pmol/mL (3.85SD) *p* = 0.010, Figure 5B). Seminal/serum sLHCGR-ratio was also inversely associated to sperm concentration (β −1.4 *p* = 0.014, Figure 5B) and men with sperm concentration below 15 mill sperm/mL (WHO reference level) had significantly higher seminal/serum sLHCGR-ratio than those with normal sperm concentration (mean 4.17 pmol/mL (7.34SD) vs. 1.41 pmol/mL (3.48SD) *p* = 0.006, Figure 5B).

### 3.9. sLHCGR Bound to hCG in Serum Is Unchanged after Injection of 5000 IU Hcg

**Figure 6:**
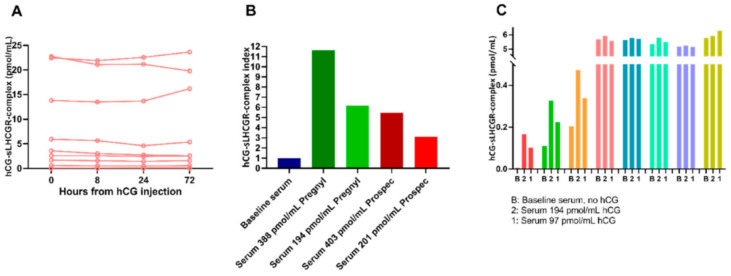
Sandwich-ELISA measuring hCG bound to sLHCGR in serum with and without intervention and proteins detectable in serum at the correct size. (**A**): The bound fraction hCG-sLHCGR before and after injection with 5000 IU hCG in 8 healthy men. (**B**): Measurement of the hCG-sLHCGR complex in a serum sample (blue: baseline), after spiking with 2 commercially available hCG products in different concentrations (green and red, respectively) (**C**): Level of the hCG-sLHCGR complex in serum from 8 infertile men after spiking with hCG (Pregnyl) in 2 concentrations.

The circulating complex of hCG bound to sLHCGR in serum was assessed prior to and after injection of hCG in eight healthy men from the clinical intervention-group. A majority of the men presented with high levels of hCG-sLHCGR already prior to hCG injection despite no detectable hCG in the serum (Figure 6A). We did not find any change in hCG-sLHCGR levels upon injection neither after 8, 24 nor 72 h (Figure 6A). We then measured hCG-sLHCGR in a serum sample from a man and subsequently added two different commercially available hCG products (Pregnyl^®^ and Prospec^®^) in two different concentrations (approximately 400 pmol/mL and 200 pmol/mL). Adding Pregnyl in high and low concentration, respectively, increased hCG-sLHCGR 12-fold and 6-fold, compared to baseline. Adding Prospec in high and low concentration, increased hCG-sLHCGR 5.5-fold and 3-fold, compared to baseline (Figure 6B). In a repeated analysis we used baseline serum from eight infertile men, adding Pregnyl in two concentrations (approximately 200 pmol/mL and 100 pmol/mL), 3 samples did show an increase in hCG-sLHCGR (Figure 6C, red, green, and orange), remaining 5 samples were saturated even prior to adding hCG and did not change upon adding hCG (Figure 6C). Finally, serum from a pregnant woman showed that she had comparable circulating hCG-sLHCGR levels as the healthy and infertile men.

## 4. Discussion

This study shows that sLHCGR identified by three different antibodies is released into body fluids and that the concentration of sLHCGR in serum and seminal fluid is linked with gonadal function in boys and men.

### 4.1. sLHCGR in Body Fluids Detected by ELISA and LC-MS/MS

The 1000-fold inter-individual variation in sLHCGR levels in serum from normal boys and men challenges an important influence of sLHCGR on LH signaling under normal conditions as neither the men with extremely low nor high concentration had any obvious reproductive or endocrine phenotypes. Technically, the applied ELISA platform produced reproducible data although with some inter-assay variability. Off-target effects are difficult to exclude, but we were unable to detect sLHCGR in mouse serum, as this peptide is species-specific, which supports the specificity since no cross-reaction was found with other mouse serum proteins. Likewise, did we not detect sLHCGR in media without human tissue/cells, but still containing FSB (fetal bovine serum). However, five freeze and thaw cycles of serum caused a 40% reduction in the measured sLHCGR concentration. This is a serious limitation to the applicability of this ELISA, but in general most adult men had stable and comparable sLHCGR levels when measured repeatedly over several years, regardless of the time of the day blood sampling or season.

The concentration of sLHCGR in the serum of men was lower than anticipated based on previous reports in women [16]. Serum sLHCGR could not be verified with standard low sensitivity LC-MS/MS despite being reported in the nanomolar range, which questions previous reports on the relatively high concentration in body fluids. Our aim with performing LC-MS/MS was solely to investigate whether sLHCGR was detectable in serum, not to make exact quantification of the precise level, therefore we did not calculate recovery percentage after depletion or use internal standard. Theoretically, the LC-MS/MS method generally detects peptides down to 10 µg/L, the heaviest LHCGR fragment detected in our samples was 75 kDa, which equivalents to a detection limit of sLHCGR in the picomolar range. Thus, it might be fair to conclude that the actual level of sLHCGR in our serum samples is even lower. However, previous work using high-sensitive LC-MS/MS on serum *has* identified LHCGR in serum, which supports the presence of LHCGR in circulation but not in the nanomolar range [18].

This study provides no firm proof of sLHCGR in serum, but detection using three different antibodies targeting different epitopes of LHCGR which recognize fragments of the expected size in serum as well as in the gonads, strongly supports that LHCGR fragments may be present in serum. Previous reports have shown that mammalian cells transfected with human LHCGR cDNA produce several LHCGR fragments of varying size [9,30,31,32] but the specific function of these LHCGR fragments remains to be established. It cannot be excluded that the used ELISA platform cross-reacts with another factor in serum in addition to LHCGR, a notion that to some extend is supported by the finding that LHCGR was not detectable in high nanomolar-micromolar concentrations with LC-MS/MS.

### 4.2. sLHCGR Bound Fraction and Dynamics

The specificity of the ELISA platform, which is based on two antibodies raised against specific epitopes of LHCGR, has previously been carefully demonstrated in vitro, but to the best of our knowledge, dynamic tests in humans have not been performed. In this study, we made numerous failed attempts to detect sLHCGR bound to hCG in serum. Injection of 5000 IU hCG into a man would in theory lead to serum levels of hCG measurements during 6–8 weeks of pregnancy. Several of our men presented with baseline values (prior to hCG injection) well above this level, which questions the specificity of this sandwich-ELISA detecting the sLHCGR-hCG bound fraction. Furthermore, we did not see any change in the level of sLHCGR-hCG-complex upon hCG injection. In our hands, the quantification of hCG-sLHCGR did not provide a reliable reproducible output even when we used spiked samples or patients treated with high dose hCG.

### 4.3. sLHCGR Is Linked with Pituitary and Gonadal Function

Our working hypothesis was that serum sLHCGR, due to its ability to bind to hCG/LH, would diminish LH action and maybe be associated with gonadal insufficiency.

We found no link with serum LH, androgen production, or hCG-induced testosterone increase, which highlights that serum sLHCGR is not predictive for LH and hCG effects on Leydig cell function.

However, we argue that there was an indirect link between serum sLHCGR and pituitary function because sLHCGR levels decreased during pubertal development and prepubertal boys had significantly higher sLHCGR than postpubertal boys. The biological explanation for the decline in sLHCGR during puberty is unknown and it remains unclear whether LH is responsible for this decrease in serum sLHCGR during puberty, but there was no decline in sLHCGR in boys with KS, which indicates that sLHCGR release in KS boys may be an early signal of gonadal insufficiency.

The inverse relationship with Inhibin/FSH ratio in healthy adult men suggests a link between high sLHCGR and poor gonadal function. Interestingly, seminal fluid sLHCGR was positively associated with serum FSH and borderline inversely associated with Inhibin B, which further supports a link with gonadal function also in infertile men. The link with the gonadal function was further supported by the strong inverse association between seminal sLHCGR and total sperm count and sperm concentration in infertile men, which suggests that LHCGR may exert a direct effect on the gonad and not exclusively indirectly through reproductive hormones.

The ratio between seminal/serum sLHCGR was significantly higher in infertile men compared with healthy men, suggesting some dependence of serum availability of sLHCGR in the reproductive tract. Moreover, seminal fluid concentration and seminal/serum sLHCGR-ratio were higher in men with poor sperm production may be due to leaky tubules or increased release, and men with severe oligospermia had significantly higher sLHCGR levels than men with higher sperm production. The rapid decrease in serum sLHCGR and suppressed urinary sLHCGR already 8 h after hCG injection indicates a direct effect as it precedes the increase in testosterone and decrease in LH levels. This indicates that LHCGR agonists may directly influence the degradation of sLHCGR and thereby modify the gonadal function in addition to their androgen stimulatory role. We did challenge the men with a supraphysiological dosage of hCG and downregulation of receptors as a result of altered pulsation-rhythm or rapidly altered serum-concentration is a known phenomenon, thus this could be part of a protective strategy as a result of sudden alterations.

### 4.4. Biological Role of sLHCGR

One may challenge the biological functionality of sLHCGR fragments in serum, but it has previously been shown that fragments of LHCGR, for instance, the extracellular part, can heterodimerize with an LHCGR containing the transmembrane-domain and intracellular region [33]. Moreover, human Granulosa cells with an LHCGR-splice variant lacking its transmembrane domain binds and inhibits the full-length LHCGR [34] and an intracellularly trapped splice variant lacking exon 9 binds FSH-receptor and inhibits its insertion into the membrane [35]. Finally, displaced LHCGR without the transmembrane-domain has been shown in malignant adrenal tumors [36]. The presence of a factor that can bind LH in serum has also been demonstrated functionally as serum from men with renal failure can bind and inhibit the action of LH [37]. The obvious question is whether all available sLHCGR fragments in serum binds LH or hCG and if the proportion of hormone bound to sLHCGR is more informative.

### 4.5. sLHCGR Origin and Target

Seminal fluid concentrations of sLHCGR were significantly lower than in serum, moreover, men with anorchia had detectable levels of sLHCGR in serum and one may speculate whether sLHCGR in serum may at least partially originate from other organs than the gonads [38]. It has been shown that *LHCGR* is expressed in several non-gonadal tissues already during fetal life [39]. We corroborated this by showing detectable sLHCGR in culture-media from both the human fetal adrenal gland and human fetal kidney at higher levels than in the media from TCam-2 cells where effects of both LH and hCG are evident [23]. However, three adult patients had comparable levels of sLHCGR in arterial and venous blood across both liver, kidney, and lower extremity, indicating that the soluble receptor most likely is not secreted/cleaved in large amounts from just one organ. The presence of LHCGR in body fluids and organs other than gonads, suggests non-classical effects of its ligand, LH. The existence of non-gonadal effects of LHCGR has been challenged by the lack of effects observed in LHCGR Knock out mice, but one of these models had an inactivating mutation exclusively in exon 11 and thus still a residual function of the intracellular and transmembrane domain which in theory would allow potential but controversial heterodimerization with other ligand-binding domains of other members in the same receptor-family [5].

### 4.6. Diagnostic Potential

The apparent link with gonadal function and the consistent level of sLHCGR over time enables some prognostic value, if sLHCGR in serum changes in response to disease onset or progression, while the high interindividual variability within patient groups challenges diagnostic potential.

## 5. Conclusions

In conclusion, this study shows that sLHCGR is released into serum and some body fluids. Serum levels of sLHCGR are associated with pubertal development and gonadal function and are temporarily suppressed by high levels of serum-hCG. sLHCGR in serum may have prognostic value as a marker for gonadal function and sLHCGR is released into serum by testis and other organs, which may indicate putative extra-gonadal effects of LH or hCG in boys and men.

## Figures and Tables

**Table 1:** Baseline characteristics of 11 men receiving 5000 IU of human chorionic gonadotropin of 148 young men from the general population, and of 307 infertile men. * shows median.

Cross-Sectional Analyses									
	hCG Injection Men			Young Healthy Men			Infertile Men		
	Mean	SD	*n*	Mean	SD	*n*	Mean	SD	*n*
Age (years)	31.0	8.2	11	19.1	3.1	146	34.8	6.6	330
BMI (kg/m2)	23.1	2.1	11	22.1	2.7	142	26.4	4.4	301
LH serum (IU/L)	3.3	1.6	11	4.1	1.8	148	4.4	2.4	301
FSH serum (IU/L)	4.0	2.7	11	3.6	2.7	148	5.3	4.2	301
Testosterone serum (nmol/L)	19.6	3.6	11	17.7	6.5	148	14.4	4.6	301
Estradiol serum (pmol/L)	88.0	27.0	11	84.7	26.5	148	100.3	26.8	301
SHBG serum (nmol/L)	29.4	8.5	11	28.4	12.3	148	30.68	12.1	301
Inhibin B serum (pg/mL)	192.4	66.1	11	182	75	148	159	78	301
Inhibin B/FSH-ratio serum	96.9	90.6	11	88	106	148	55	65	301
AMH serum (pmol/L)	46.5	17.6	11	-	-	-	40	26	301
sLHCGR serum (pmol/mL)	0.61	1.08	11	2.7	6.4	148	1.05	3.15	297
sLHCGR urine (pmol/mL)	0.024	0.017	11	-	-	-	-	-	-
sLHCGR seminal fluid (pmol/mL)	-	-	-	0.10	0.24	59	0.05	0.08	61
Testicular size (mL)	-	-	-	13.3	4.0	144	12.7	4.2	275
Semen volume (mL)	-	-	-	3.4	1.3	148	3.9	1.8	330
Total sperm count * (mill)	-	-	-	140	125	146	96	127	329
Sperm concentration * (mill/mL)	-	-	-	40	43	146	27	33.9	330
Progressive motile sperm (%)	-	-	-	58	17	146	32	19.8	315
Morpholgical normal sperm (%)	-	-	-	7.6	4.3	145	3.3	2.9	320

**Table 2:** Baseline characteristics of 36 healthy boys followed longitudinally. LOD = limit of detection. * all but two measurements were below LOD.

Longitudinal Measurements of Peripubertal Boys						
	First Examination			Last Examination		
	Pre-Pubertal			Post-Pubertal		
	Mean	SD	*n*	Mean	SD	*n*
Age (years)	9.11	0.96	36	14.79	1.44	36
BMI (kg/m2)	17.32	2.1	36	20.37	3.6	36
Testicular size (mL)	2.1	0.4	36	15.7	5.5	36
sLHCGR (pmol/mL)	1.36	2.21	34	0.56	0.83	32
LH (IU/L)	0.090	0.12	36	2.85	1.28	36
FSH (IU/L)	0.79	0.45	36	3.31	1.44	36
Testosterone (nmol/L)	<LOD	-	36	14.34	4.83	36
Androstenedione (nmol/L)	1.07	1.19	36	3.43	1.58	7
DHEASO4 (µg/L)	1.58	1.15	36	2.58	1.16	7
Estradiol (pmol/L)	<LOD *	-	36	61.39	28.52	36
SHBG (nmol/L)	124	41	36	43.18	27.00	36
Inhibin B (pg/mL)	92	32	36	218.53	72.54	36
Inhibin B/FSH-ratio	164	107	36	90	72	36
IGF-1 (µg/L)	182	65	36	442	137	16
IGF-BP3 (µg/L)	3529	786	36	4981	723	16
Urinary LH (IU/L)	0.53	1.15	35	3.18	2.18	28
Age at pubertal onset, range (years)		9.86–13.73				
Age at pubertal onset, mean (years)		11.41				
Number of examinations, range		5–6				
Age at examination, range (years)		6.4–16.4				

**Table 3:** Baseline characteristics of 63 boys and men with Klinefelter Syndrome, seven boys were further followed longitudinally. LOD = limit of detection.

Cross Sectional Analysis of Men with Klinefelter Syndrome						
	Pre-Pubertal			Post-Pubertal		
	Mean	SD	*n*	Mean	SD	*n*
Age (years)	6.53	3.48	16	23.29	10.26	47
sLHCGR (pmol/mL)	1.32	1.14	16	0.92	2.55	47
LH (IU/L)	0.10	0.13	16	10.24	8.43	47
FSH (IU/L)	0.63	0.38	15	21.39	21.34	26
Testosterone (nmol/L)	<LOD	-	16	11.89	5.74	47
Estradiol (pmol/L)	<LOD	-	16	60.13	33.80	47
SHBG (nmol/L)	125	40	16	35	18	47
Inhibin B (pg/mL)	93	66	5	32	47	11
Inhibin B/FSH-ratio	173	156	5	13	26	11
AMH (pmol/L)	902	420	15	72	132	38
**KS boys followed longitudinally (*n* = 7)**						
Age at pubertal onset, range (years)		9.60–12.85				
Age at pubertal onset, mean (years)		11.76				
Number of examinations, range		3–6				
Age at examination, range (years)		7.44–16.10				

## Data Availability

The data presented in this study are available on request from the corresponding author. The data are not publicly available due to privacy restrictions.

## Supplementary Materials

The following are available online at https://www.mdpi.com/2072-6694/13/6/1329/s1, Figure S1: Immunohistochemistry and RT-PCR, Figure S2: Testosterone and LH, Figure S3: Inhibin B/FSH-ratio, Table S1: LC-MS/MS proteins.
