# Supplementary material for: Possible Relevance of Soluble Luteinizing Hormone Receptor during Development and Adulthood in Boys and Men
Source: Cancers (Basel). 2021 Mar 16;13(6):1329. doi: 10.3390/cancers13061329 (PMC7999540; doi:10.3390/cancers13061329)
Supplement: Supplementary file 1 [file cancers-13-01329-s001.zip › SUP-PUB/cancers-1152136-supplementary -for PUB.pdf]

cancers

MDPI

Article

Possible Relevance of Soluble Luteinizing Hormone Receptor during Development and Adulthood in Boys and Men

Li Juel Mortensen, Mette Lorenzen, Anne Jørgensen, Jakob Albrethsen, Niels Jørgensen, Søren Møller, Anna-Maria Andersson, Anders Juul and Martin Blomberg Jensen

Supplementary figures and table

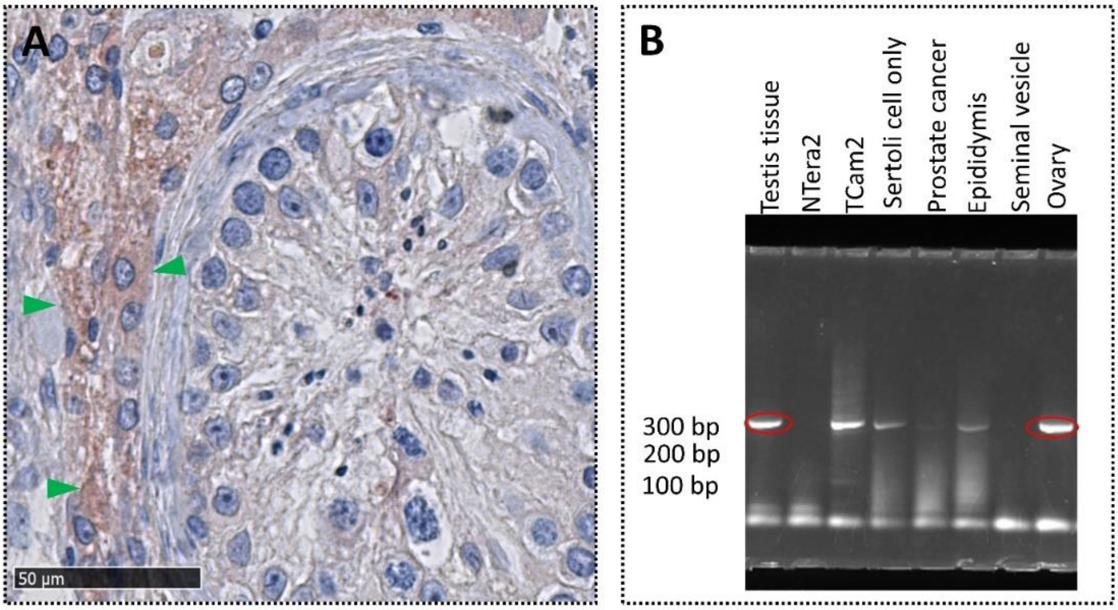

Figure 1. Expression of sLHCGR. (**A**): Immunohistochemistry of human adult testis tissue stained with Santa Cruz Biotechnology (SC-25828) shows expression of LHCGR in the membrane and cytoplasm of the Leydig cells (green arrows). Image is from × 40 magnification. (**B**): RT-PCR with a primer spanning exon 11 of LHCGR in samples of testis tissue, adjacent reproductive organs and cell lines. The band of approximately 300 bp marked with red circles were sequenced and proved to be LHCGR.

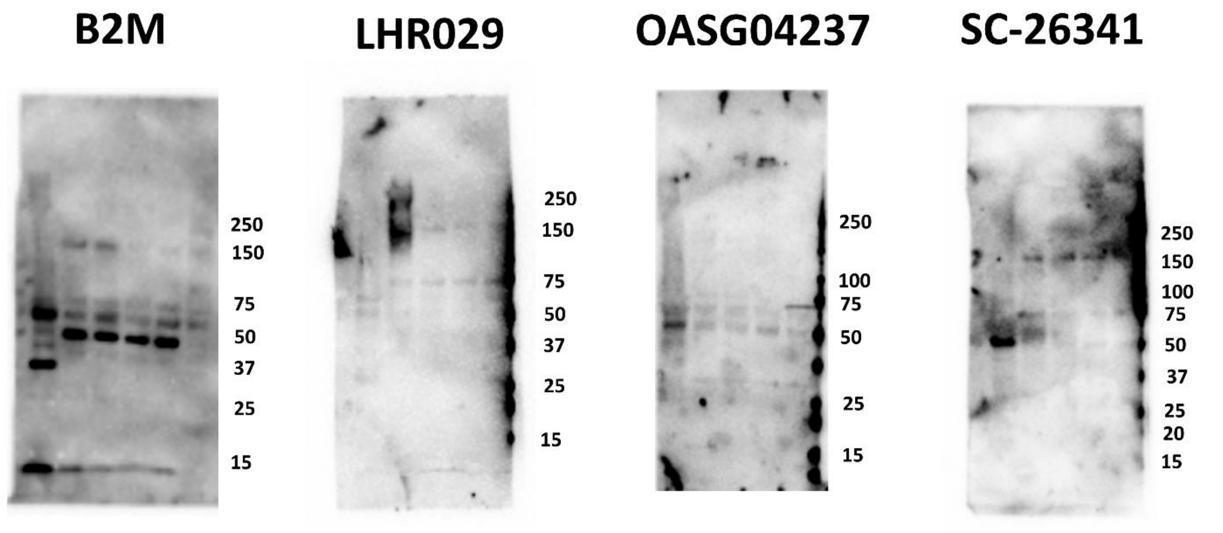

		Testis tissue	Serum high sLHCGR	Serum low sLHCGR
12 kDa	**B2M**	4144	1797	1226
75 kDa	**LHR029**	NA	2126	2095
	**OASG04237**	973	495	498
	**SC-26341**	NA	1446	400
50 kDa	**LHR029**	1445	870	506
	**OASG04237**	851	351	366
	**SC-26341**	1462	NA	66

Figure 2. Uncropped Western Blot using three different antibodies targeting LHCGR. Loading from left: testis tissue, serum from healthy man with high sLHCGR, serum from healthy man with low sLHCGR, serum from man with testicular cancer (seminoma), serum from man with testicular cancer (non-seminoma). Table shows densitometry readings performed in Image-J with measured peak intensity and calculated area under the peak.

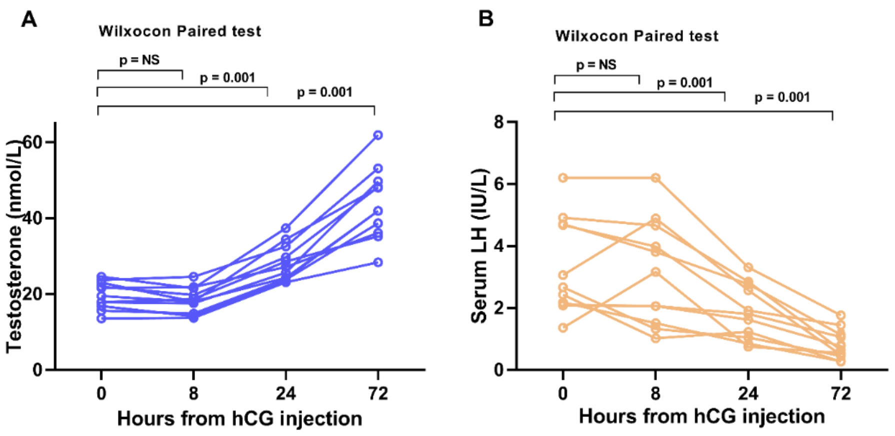

Figure 3. Eleven healthy men injected with 5000 IU hCG (**A**): Changes in testosterone after 8, 24 hours and 72 hours. (**B**): Changes in LH after 8, 24 hours and 72 hours. After correcting for multiple comparison using Bonferroni: level of significance <0.0167.

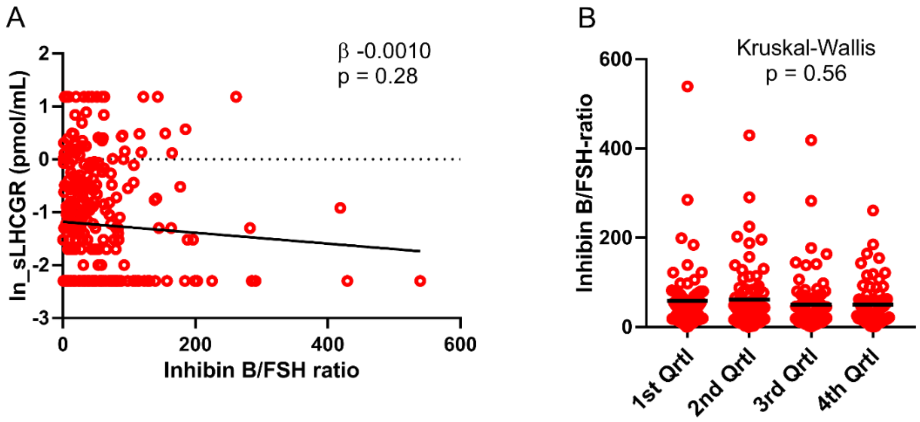

Figure 4. No significant association between sLHCGR and Inhibin B/FSH-ratio in 297 infertile men. (**A**): Crude association (**B**): sLHCGR split in quartiles, with a downward trend in Inhibin B/FSH ratio, but not this was not statistically significantly different between groups.

Table 1. Table shows proteins in serum from a pregnant woman, depleted of albumin and IgG and analyzed by LC-MS/MS. SDS- gel electrophoresis fragments of 35 kDa, 50 kDa, 60 kDa and 75 kDa were included. The Mascot Score is a statistical score that reflects how the MS data match the database. The higher the Mascot score the higher the confidence of a protein being identified. High abundant protein will often produce relatively high Mascot scores. The Mascot score is above the 95% confidence level for all the reported proteins in this table. We did not detect sLHCGR by LC-MS/MS in the current sample which could be due to low serum-concentrations.

**Sample A: 35 KD**		**Sample B: 50 KD**	
**Protein Name**	**Mascot Database**	**Protein Name**	**Mascot database**
complement C 3	1857	albumin	2303
alpha-1-antitrypsin	1728	vitamin D-binding protein	1741
apolipoprotein A-I	1375	complement C3	1592
albumin	1145	alpha-1 antitrypsin	1246
ceruplasmin	1068	ceruplasmin	1032
prothrombin	857	serotranferrin	881
serotransferrin	846	apolipoprotein A-I	852
immunoglobulin kappa	772	haptoglobin	830
haptoglobin	692	leucine-rich alpha-2-glycoprotein	785
immunoglobin lamda-1 light chain	597	complement C4-A	688
inter-alpha-trypsin inhibitor heavy chain H4	597	sex-hormone binding globulin	633
apolipoprotein D	552	paraoxonase	446
immunoglobulin lambda constant 2	543	interalpha-trypsin inhibitor heavy chain H4	423
apolipoprotein B-100	501	alpha-2-HS-glycoprotein	416
inter-alpha-trypsin inhibitor heavy chain H2	475	clusterin	377
angiotensinogen	473	prothrombin	359
vitamin D-binding protein	431	angiotensinogen	323
complement C4-A	425	alpha-2-antiplasmin	320
clusterin	407	kininogen-1	304
transthyretin	310	Ig alpha-1 chain C region	297
apolipoprotein A-II	263	alpha-1-acid glycoprotein	288
immunoglobulin kappa vairabel 3-20	259	zinc-alpha-2-glycoprotein	281
complement C5	226	alpha-1-acid glycoprotein 2	260
paraoxonase	220	hemopexin	256
immunoglobulin kappa variable 2D-28	205	apolipoprotein A-II	233
alpha-1B-glycoprotein	198	alpha-2-macroglubolin	210
apolipoprotein M	192	immunoglubulin heavy constant gamma 2	192
amyloid P-component	190	antothrombin-III	188
galactin-3-binding protein	174	immunoglobulin gamma-1 heavy chain	167
coagulations factor xII	155	beta-2 glycoprotein 1	154
apolipoprotein E	151	alpha-1B-glycoprotein	151
immunoglobulin gamma 1 heavy chain	148	immunoglubulin heavy constant mu	90
immunoglobulin lambda variable 3-9	144		
immunoglobulin kappa variable 1-5	133		
protein AMBP	126		
immunoglobulin lambda variable 1-47	114		
immunoglubulin J chain	75		
hemoglobin subunit beta-A	64		

Sample C: 60 KD		Sample D: 75 KD	
**Protein Name**	**Mascot Database**	**Protein Name**	**Mascot Database**
albumin	1411	serotransferrin	4334
alpha-1-antitrypsin	680	albumin	3859
serotransferrin	528	ceruplasmin	1700
vitamin D-binding protein	520	complement C3	1402
albumin	401	alpha-1B-glycoprotein	1290
immunoglobulin heavy constant alpha 1	314	alpha-1-antitrypsin	1106
inter-alpha-trypsin inhibitor heavy chain H4	285	hemopexin	858
complement C3	211	immunoglobulin heavy constant alpha 1	702
ceruplasmin	209	apolipoprotein A-I	505
immunoglobulin gamma-1 heavy chain	146	immunoglobulin heavy constant mu	500
vitronectin	94	alpha-2-macroglobulin	498
		kininogen-1	451
		afamin	355
		haptoglobin	328
		inter-alpha-trypsin inhibitor heavy chain H4	297
		alpha-1-antichymotrypsin	279
		lumican	272
		angiotensinogen	256
		apolipoprotein A-II	228
		complement C4-A	184
		apolipoprotein B-100	174
		immunoglobulin gamma-1 heavy chain	154
